# Expanded roles of lactate-sensing LldR in transcription regulation of the *Escherichia coli* K-12 genome: lactate utilisation and acid resistance

**DOI:** 10.1099/mgen.0.001015

**Published:** 2023-05-23

**Authors:** Takumi Anzai, Kaede Kijima, Miki Fujimori, Soma Nakamoto, Akira Ishihama, Tomohiro Shimada

**Affiliations:** ^1^​ School of Agriculture, Meiji University, Kawasaki, Kanagawa, Japan; ^2^​ Micro-Nano Technology Research Center, Hosei University, Koganei, Tokyo, Japan

**Keywords:** Transcription factor, LldR, lactate, acid resistance, Genomic SELEX (gSELEX), *Escherichia coli*

## Abstract

LldR is a lactate-responsive transcription factor (TF) that transcriptionally regulates the *lldPRD* operon consisting of lactate permease and lactate dehydrogenase. The *lldPRD* operon facilitates the utilisation of lactic acid in bacteria. However, the role of LldR in whole genomic transcriptional regulation, and the mechanism involved in adaptation to lactate remains unclear. We used genomic SELEX (gSELEX) to comprehensively analyse the genomic regulatory network of LldR to understand the overall regulatory mechanism of lactic acid adaptation of the model intestinal bacterium *Escherichia coli *. In addition to the involvement of the *lldPRD* operon in utilising lactate as a carbon source, genes related to glutamate-dependent acid resistance and altering the composition of membrane lipids were identified as novel targets of LldR. A series of *in vitro* and *in vivo* regulatory analyses led to the identification of LldR as an activator of these genes. Furthermore, the results of lactic acid tolerance tests and co-culture experiments with lactic acid bacteria suggested that LldR plays a significant role in adapting to the acid stress induced by lactic acid. Therefore, we propose that LldR is an l-/d-lactate sensing TF for utilising lactate as a carbon source and for resistance to lactate-induced acid stress in intestinal bacteria.

## Data Summary

The gSELEX data of LldR were deposited in to the ‘Transcription factor profiling of *Escherichia coli *’ (TEC) database at the National Institute of Genetics (https://shigen.nig.ac.jp/ecoli/tec/).

**Impact Statement**

Lactic acid is produced by a range of gut bacteria, including traditional probiotic species such as lactobacilli. As a result, other gut bacteria are thereby exposed to this acid. Clearly, these other bacteria persist in the gut; however, the exact mechanism by which they adapt to the acid stress induced by lactic acid remains unclear. By using the gSELEX-chip method, we identified the genomic regulatory role of the lactate-responsive TF LldR as an activator of genes involved in utilising lactate as a carbon source, glutamate-dependent acid resistance and altering the composition of membrane lipids. In fact, LldR was found to play an important role in the growth of *E. coli* in lactate tolerance and co-culture with lactic acid bacteria. The results of this study provide insight into the molecular mechanisms of intestinal bacterial adaptation to lactic acid and lactic acid-producing bacteria.

## Introduction

Lactate is a significant product of anaerobic carbon metabolism that also serves as a carbon and energy source for both anaerobic and aerobic microorganisms. *Escherichia coli* have an *lldPRD* operon (formerly labelled *lct*) responsible for aerobic l-lactate metabolism [1] and can utilise l-lactate as the sole source of carbon [2]. The operon consists of three genes that form a single transcriptional unit inducible by l- or d-lactate. The *lldD* gene encodes dehydrogenase, *lldP* encodes permease, and *lldR* encodes a TF [1]. l-Lactate is also recognised by the permease encoded by *glcA*, but this gene is not induced by growth on l-lactate, indicating that LldP mediates l-lactate uptake *in vivo* [3, 4].

Transcriptional regulation of *E. coli* in the presence of lactate is mediated by the lactate-sensing TF LldR, which belongs to the GntR-family, and senses l-lactate and d-lactate [3]. The *lldPRD* operon is activated by LldR [1, 5] and the two-component regulatory system ArcAB [6, 7], which functions under anaerobic conditions, and pyruvate-sensing TF PdhR [8]. Only this *lldPRD* operon has been reported as a target of LldR in RegulonDB [9, 10, https://regulondb.ccg.unam.mx/].

Thus, *E. coli* can use lactic acid as a carbon source, under acid stress induction [11]. Weak organic acids, including lactic acid, are more potent against bacteria, especially under mildly acidic conditions when the acid groups are more likely to be protonated [12]. At low pH, the protonated form of a weak acid (neutral) diffuses through the cell membrane and dissociates intracellularly, thereby lowering the intracellular pH [13]. Dissociated anions accumulate within the cell and can cause turgor stress due to increased osmotic pressure [14]. The stress of short-chain organic acids, including lactic acid, on *E. coli* K-12 strain in the stationary phase has been demonstrated using cell viability and transcriptome analysis [15]. Genes involved in oxidative stress, cell envelope, cold shock stress, and iron and manganese uptake were upregulated as a common response to hydrochloric, acetic, and lactic acid stress. Although the effects of and response to lactate have been analysed previously, in terms of general acid stress, this has not been distinguished from the lactate-specific stress response, and the mechanism remains unclear.

Lactic acid-producing bacteria such as lactobacilli are often used as probiotics [16, 17]. One potentially beneficial effect of these bacteria is that they release lactic acid, which may inhibit the growth of harmful bacteria by lowering the pH, thereby helping to prevent intestinal putrefaction and normalising the intestinal microbiota [18–20]. Multiple acid resistance (AR) systems have been described for *E. coli* [21, 22]; AR1 is an oxidative AR system repressed by glucose that is σ^S^-dependent and does not require an externally derived amino acid. AR2 (also called the GAD system) is dependent on glutamate, AR3 on arginine, AR4 on lysine, and ODAR on ornithine [23]. Alteration of cell envelope components, such as membrane proteins, fatty acids, chaperones, and proton-consuming systems, has also been reported to be effective in acid stress resistance in *E. coli* [24]. Although lactate causes various cellular effects via acid stress, the only reported target of the lactate-responsive TF LldR is the *lldPRD* operon for the utilisation of lactate. Lactate-specific acid stress adaptation has not been reported, and a comprehensive genome-wide analysis has not been conducted. Analysis of the genomic regulatory network of lactate-sensing TFs may reveal physiological roles of lactate other than simply as a carbon source. The gSELEX method was developed to elucidate the genomic regulatory networks of TFs based on the identification of the binding site(s) *in vitro* of the test TF in the *E. coli* genome [25, 26]. The gSELEX method has successfully revealed new functions even for known TFs [8, 27–36]. In this study, we sought to understand the overall lactate response mechanism of *E. coli *; using gSELEX screening to analyse the entire genomic regulatory network of LldR.

## Methods

### Bacterial strains and plasmids

*Escherichia coli* K-12 W3110 type-A [37] was used as the DNA source to construct the LldR expression plasmid. The *E. coli* K-12 W3110 type-A genome was used to construct the DNA library required for gSELEX screening. *E. coli* DH5α cells were used for plasmid amplification, and *E. coli* BL21 (DE3) cells were used for LldR expression. *E. coli* BW25113 [38], its *lldR* single-gene knockout mutant [39], and the expression plasmid from the ASKA clone library were obtained from the *E. coli* Stock Centre (National Bio-Resource Centre, Chiba, Japan). Plasmid pPET21 was used to construct the LldR expression plasmid, pLldR. Cells were grown in M9 minimal medium [40], supplemented with casamino acids (CAA) (0.2 %) at 37 °C with constant shaking at 150 r.p.m. When necessary, kanamycin (20 μg ml^−1^) or chloramphenicol (30 μg ml^−1^) was added to the medium. Cell growth was monitored by measuring the turbidity at 600 nm (OD_600_).

### Purification of LldR protein

The construction of the plasmid (pLldR) for expressing LldR and procedures for expression and purification followed previously described procedures [25]. Briefly, LldR coding sequences were PCR-amplified using the *E. coli* K-12 W3110 genomic DNA as a template and inserted into the pET21a (+) vector (Novagen, Darmstadt, Germany) between NdeI and NotI restriction sites. The expression plasmid, pLldR, was transformed into *E. coli* BL21 (DE3) cells. Transformants were grown in LB medium, and LldR expression was induced using IPTG in the middle of the exponential phase. LldR protein was purified by affinity purification using a Ni-nitrilotriacetic acid (NTA) agarose column. The affinity-purified LldR protein was stored and frozen in the storage buffer at −80 °C until further use. Protein purity was greater than 95 %, as determined using SDS-PAGE.

### Genomic SELEX (gSELEX) screening of LldR-binding sequences

gSELEX screening was performed as previously described [25, 26]. Briefly, a mixture of DNA fragments from the *E. coli* K-12 W3110 genome was prepared by sonicating purified genomic DNA and cloned into a multi-copy plasmid, pBR322. For each gSELEX screening, the DNA mixture was regenerated using PCR. DNA fragments (5 pmol) and His-tagged LldR (10 pmol) were mixed in a binding buffer (10 mM Tris-HCl, pH 7.8 at 4 °C, 3 mM magnesium acetate, 150 mM NaCl, and 1.25 mg ml^−1^ bovine serum albumin). The complex of the His-tagged LldR and the DNA fragments was purified using Ni-NTA columns. The SELEX cycle was repeated thrice to enrich the LldR-binding sequences. Mapping of SELEX fragments along the *E. coli* genome was performed using a gSELEX-chip system with a 43450-feature DNA tiling array [41]. The gSELEX sample obtained using LldR was labelled with Cy3, whereas the original genomic DNA library was labelled with Cy5. Following the hybridisation of the samples to the DNA tiling array (Agilent Technology, Santa Clara, California, USA), the Cy3/Cy5 ratio was measured, and the peaks of the scanned patterns were plotted against the positions of the DNA probes along the *E. coli* K-12 genome.

### Gel shift assay

The gel shift assay was performed according to a standard procedure [28]. Probes for LldR-binding target sequences were generated by PCR amplification using a pair of primers (Table S1a, available in the online version of this article) and Ex Taq DNA polymerase (TaKaRa, Kyoto, Japan). A mixture of each probe and LldR was incubated at 37 °C for 30 min in the binding buffer. After adding effectors, the mixture was incubated for an additional 30 min. After adding the DNA-loading solution, the mixture was subjected to 5 % PAGE. DNA in the gels was stained with GelRed (Biotium, Fremont, California, USA) and detected using LuminoGraph I (Atto, Tokyo, Japan).

### Consensus sequence analysis

A set of LldR-binding sequences, identified using the gSELEX-chip, was analysed with the MEME SUITE programme [42] to evaluate the LldR-binding sequence. Subsequently, the sequences were aligned, and a consensus sequence logo was created using WEBLOGO (http://weblogo.berkeley.edu/logo.cgi).

### RT-qPCR analysis

RT-qPCR analysis was performed according to standard procedures [43]. *E. coli* cells were inoculated into M9 minimal medium supplemented with CAA (0.2 %) at 37 °C with aeration by constant shaking at 150 r.p.m. Total RNA was extracted from exponential phase *E. coli* cells (OD_600_=0.4) using ISOGEN solution (Nippon gene, Tokyo, Japan). Total RNA was transcribed to cDNA with random primers using the THUNDERBIRD SYBR qPCR RT Set (TOYOBO, Osaka, Japan). Quantitative PCR (qPCR) was conducted using THUNDERBIRD SYBR qPCR Mix (TOYOBO) and a LightCycler 96 system (Roche, Basel, Switzerland). The primer pairs used are described in Table S1b. The cDNA templates were serially diluted four-fold and used in qPCR. The qPCR mixtures, containing 10 µl of THUNDERBIRD SYBR qPCR Mix (TOYOBO), 1 µl of each primer (5 µM stock), 7 µl of water, and 1 µl of cDNA, were ampliﬁed under the following thermal cycling conditions: 2 min at 95 °C, 45 cycles of 10 s at 95 °C and 20 s at 55 °C, and then incubated for 20 s at 72 °C. The 16S rRNA expression level was used to normalise the varying levels of the test samples, and the relative expression levels were quantiﬁed using Relative Quantification software provided by Roche. The results are presented as the average of three independent experiments.

### Reporter assay of *lldP* promoter activity

The *lldP* promoter fragment, approximately 400 bp in length between the initiation codon and upstream sequence, was amplified by PCR using a pair of primers (Table S1c) and cloned into the pRS551 plasmid vector. Single-copy *lacZ* (*β*-galactosidase) reporter strains containing *lldP* promoter-lacZ were constructed using λ the RS45 phage vector, as described previously [44]. The recombinant phage-containing *lldP* promoter-*lacZ* fusion was isolated from the resulting phage lysate and used to infect *E. coli* BW25113 and JW3579 strains lacking a Km marker for screening kanamycin resistance. Single-copy *lldP* promoter-*lacZ* fusion strains were grown in M9-CAA (0.2 %) medium supplemented with 20 mM of each organic acid, and *β*-galactosidase activity was measured using ONPG as a substrate, as described previously [8].

### Northern blot analysis

Total RNA was extracted from exponential phase *E. coli* cells (OD_600_=0.4) using ISOGEN solution (Nippon Gene). RNA purity was verified by electrophoresis on a 1.5 % agarose gel with formaldehyde, followed by staining with GelRed. Northern blot analysis was performed as previously described [33]. Dig-labelled probes were prepared by PCR amplification using W3110 genomic DNA (50 ng) as a template with a pair of primers (Table S1d), DIG-11-dUTP (Roche), dNTP, gene-specific forward and reverse primers, and Ex Taq DNA polymerase. Total RNA (3 µg) was incubated in formaldehyde-morpholinepropanesulfonic acid (MOPS) gel-loading buffer for 10 min at 65 °C for denaturation, subjected to electrophoresis on formaldehyde-containing 1.5 % agarose gel and then transferred onto a nylon membrane (Roche). Hybridisation was performed using DIG easy Hyb system (Roche) at 50 °C overnight with a Dig-labelled probe. The membrane was treated with anti-DIG-AP Fab fragments, and CDP-Star (Roche) to detect the DIG-labelled probe and the image was scanned using LuminoGraph I (Atto).

### l-Lactate sensitivity test

*E. coli* cells were inoculated in the M9 medium with 0.2 % CAA supplemented with 20 mM of l-lactate for pre-induction at 37 °C with aeration by constant shaking at 150 r.p.m. until the OD_600_ reached 0.3, then the cultured cells were subjected to M9 medium with 0.2 % CAA in the absence or presence of 80 mM or 120 mM of l-lactate for 30 min. After the l-lactic acid challenge, the cells were spread on LB plates and inoculated overnight. The colonies formed were counted, and cell viability was calculated as the ratio of colonies formed with/without l-lactate. The relative level of sensitivity represents the mean±standard deviation (SD) of four experiments.

### Co-culture of *E. coli* and *L. plantarum*

*E. coli* and *Lactiplantibacillus plantarum* (ATCC14917) were cultured overnight in LB medium and MRS medium (Difco), respectively. Cultured *E. coli* and *L. plantarum* were diluted to 3×10^6^ and 3×10^7^ c.f.u. ml^−1^, respectively, in a sterile MRS medium, which equally supported the growth of *E. coli* and *L. plantarum *. Then, *E. coli* and *L. plantarum* were inoculated together in fresh MRS medium to a final volume of 15 ml in tightly capped 15 ml plastic tubes with static cultivation. Pure culture samples (1 ml) and co-culture samples (1 ml) were collected over 24 h of incubation to evaluate the number of viable cells. The samples were multistep diluted and spread on LB and MRS agar plates. Both plates were kept at 37 °C overnight, but to distinguish colony formation between *E. coli* and *L. plantarum *, the LB agar plates for *E. coli* were incubated under aerobic conditions, and MRS agar plates for *L. plantarum* were incubated under anaerobic conditions using an AneroPack (Mitsubishi gas chemistry). The pH of the medium was measured.

## Results

### Search for LldR-binding locations by gSELEX screening

To identify LldR binding sequences from the entire genome, we performed gSELEX screening [25, 26]. Briefly, purified His-tagged LldR was mixed with a collection of *E. coli* genome fragments (200–300 bp in length). Then, LldR-bound DNA fragments were affinity-isolated. The original substrate mixture of the genomic DNA fragments formed smear bands on PAGE. After three cycles of gSELEX, DNA fragments with a high affinity for LldR were enriched, forming sharper bands on PAGE gels (Fig. S1).

**Fig. 1.**
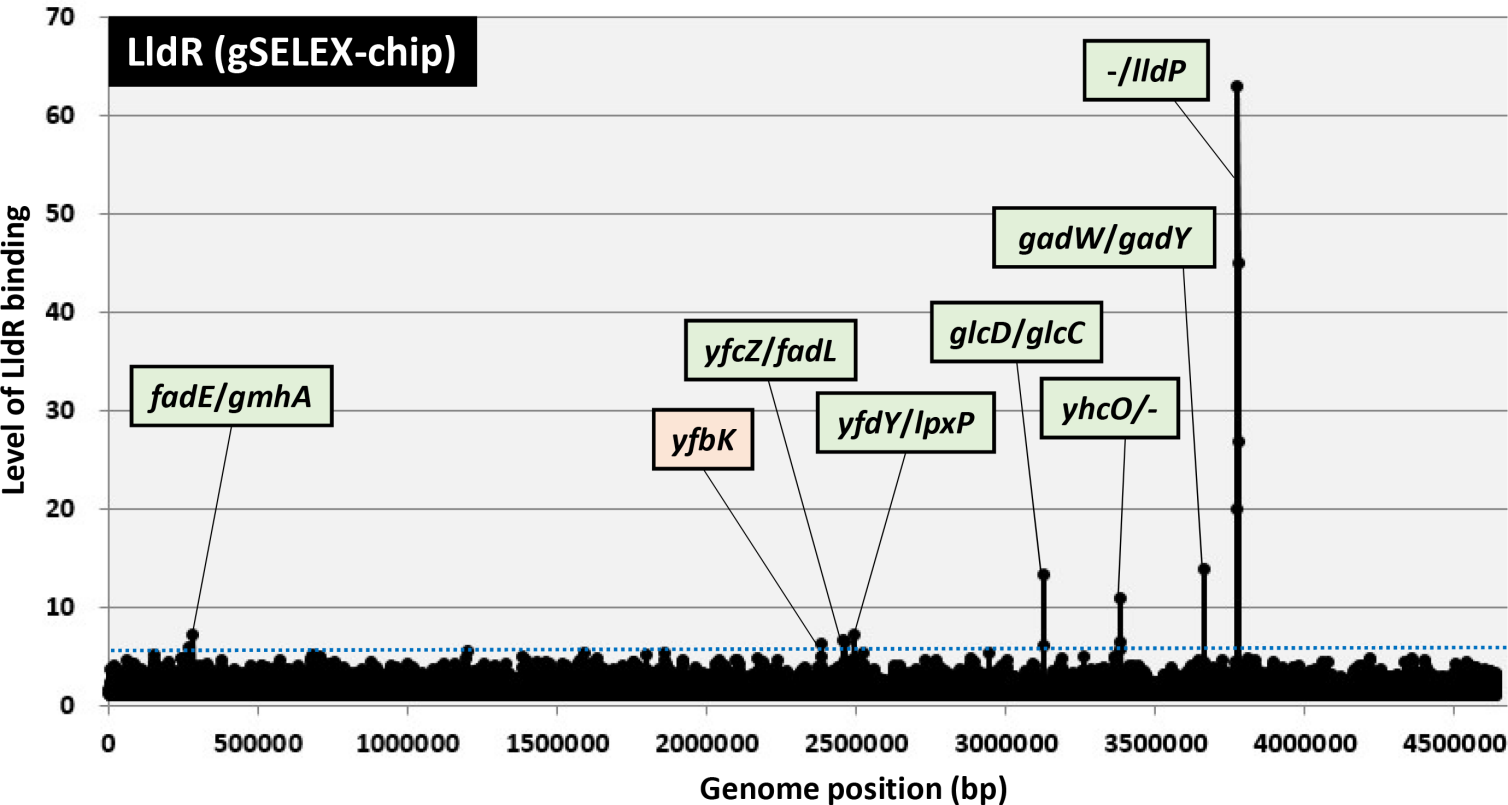
Identification of LldR-binding sites on the *E. coli* K-12 genome using gSELEX-chip. gSELEX screening of DNA-binding sequences was performed using purified C-terminal His-tagged LldR and a library of DNA segments from the *E. coli* K-12 W3110 genome. Following gSELEX, a collection of DNA fragments was subjected to gSELEX-chip analysis using the tiling array of the *E. coli* K-12 genome. A blue dotted line shows the cut-off level of 6, and the list of all LldR-binding sites from setting this cut-off level is given in Table 1. Peaks in green represent the LldR-binding sites inside spacer regions, whereas peaks shown in orange represent the LldR-binding sites inside ORFs.

DNA fragments enriched by LldR were labelled with Cy3 and the original genomic DNA library with Cy5. A mixture of fluorescently labelled samples was hybridised onto an *E. coli* DNA tiling microarray [41]. The fluorescence intensity ratio bound to each probe between the LldR sample and the original DNA library was measured and plotted against the corresponding position along the *E. coli* K-12 genome. The extent of LldR binding correlates with its affinity for LldR protein. Based on the genomic location of the peaks obtained from this result, whether they were on the promoter region or ORF, and the function of the target gene, a cutoff level of 6 was set, and eight peaks were identified in the gSELEX-chip pattern (Fig. 1). The highest peak was identified in the upstream region of the *lldP* gene, which is the promoter region of the *lldPRD* operon and the only known target of LldR. Because prokaryotic TF-binding sites are located upstream of the regulatory target genes [45, 46], 12 genes (*fadE*, *gmhA, yfcZ, fadL, yfdY, lpxP, glcD, glcC, yhcO, gadW, gadY,* and *lldP*) were predicted to be potential regulatory targets of LldR (Table 1). Within these targets, *glcD* and *lldP* form the *glcDEFGBA* operon and *lldPRD* operon, respectively (Table 1).

**Table 1.** LldR-binding sites on the *E. coli* genome gSELEX was used to search for the binding sites in LldR. Eight binding sites were identified by setting the cutoff level to 6. Column ‘LldR site’ shown in green represent the LldR-binding sites inside spacer regions, whereas those shown in orange represent the LldR-binding sites inside ORFs. Column ‘D’ shows the direction of the transcription unit. The potential target genes or operons of LldR were predicted based on adjacent genes and gene orientation (shown with bold words). Grey shading indicates genes that are not potential targets. Consensus sequences explored in each target region using MEME Suite and their conservation are shown on the right side.

												LldR box: AAnTGGTCnGACCAnTT	
	**Peak position (bp**)	**LldR Binding intensity**	**Function**	**Operon**	**Gene**	**D**	**LldR**	**D**	**Gene**	**Operon**	**Function**	**Consensus-1**	**Consensus-2**
**1**	**2 43 432**	**7.3**	**acyl coenzyme A dehydrogenase**	***fadE***	***fadE***	**<**		**>**	***gmhA***	***gmhA***	**d-sedoheptulose 7-phosphate isomerase**	**AggaGGTCtGACCAcTT(12/14**)	**tccTGGTCatAgCAccT(9/14**)
**2**	**23 83 166**	**6.4**			*elaD*	>	*yfbK*	>	*yfbL*			**tAtTGGTCaaAgaAcTT(10/14**)	**tAgaGGcCgGAatAgaT(8/14**)
**3**	**24 59 264**	**6.6**	**conserved protein**	***yfcZ***	***yfcZ***	**<**		**>**	***fadL***	***fadL***	**long-chain fatty acid outer membrane transporter**	**AgcTGGTCcGACCtaTa(11/14**)	**cAcTGGTCtGAtttcTa(9/14**)
**4**	**24 93 572**	**7.2**	**predicted inner membrane protein**	***yfdY***	***yfdY***	**<**		**>**	***lpxP***	***lpxP***	**palmitoleoyl-acyl carrier protein (ACP)-dependent acyltransferase**	**tAtaGGTCgGACCAgcT(11/14**)	**AAtTGGTtgGgcCAtca(9/14**)
**5**	**31 26 270**	**13.3**	**glycolate oxidase subunit, FAD-linked**	***glcDEFGBA***	***glcD***	**<**		**>**	***glcC***	***glcC***	**DNA-binding transcriptional dual regulator, glycolate-binding**	**AAaTGGTCtGACCggTa(12/14**)	**cAcaGGTagGACCAaTT(11/14**)
**6**	**33 84 146**	**10.9**	**predicted barnase inhibitor**	***yhcO***	***yhcO***	**<**		<	*aaeB*			**cttTcGcCaGACCAaag(8/14**)	**AAgTGGTagcggCAgga(8/14**)
**7**	**36 62 638**	**13.8**	**DNA-binding transcriptional activator for glutamate dependent acid resistance system**	***gadW***	***gadW***	**<**		**>**	***gadY***	***gadY***	**ncRNA, positive regulator of gadW and gadX**	**tttTGGTCctACCAaaT(10/14**)	**gAacGGTCaGtgCcgTa(8/14**)
**8**	**37 75 144**	**62.9**			*yibL*	>		**>**	***lldP***	***lldPRD***	**l-lactate permease**	**AAtTGGcCctACCAaTT(12/14**)	**AAgTGGcactgCCAaTT(10/14**)

The novel target genes of LldR included functions related to acid tolerance (*gadW* and *gadY*), fatty acid metabolism, alteration of membrane composition (*fadE, fadL, gmhA,* and *lpxP*), and glycolate metabolism (*glcDEFGBA* and *glcC*). However, the functions of *yfcZ, yfdY,* and *yhcO* remain unknown.

### Confirmation of LldR-binding to the newly identified targets

**Fig. 2.**
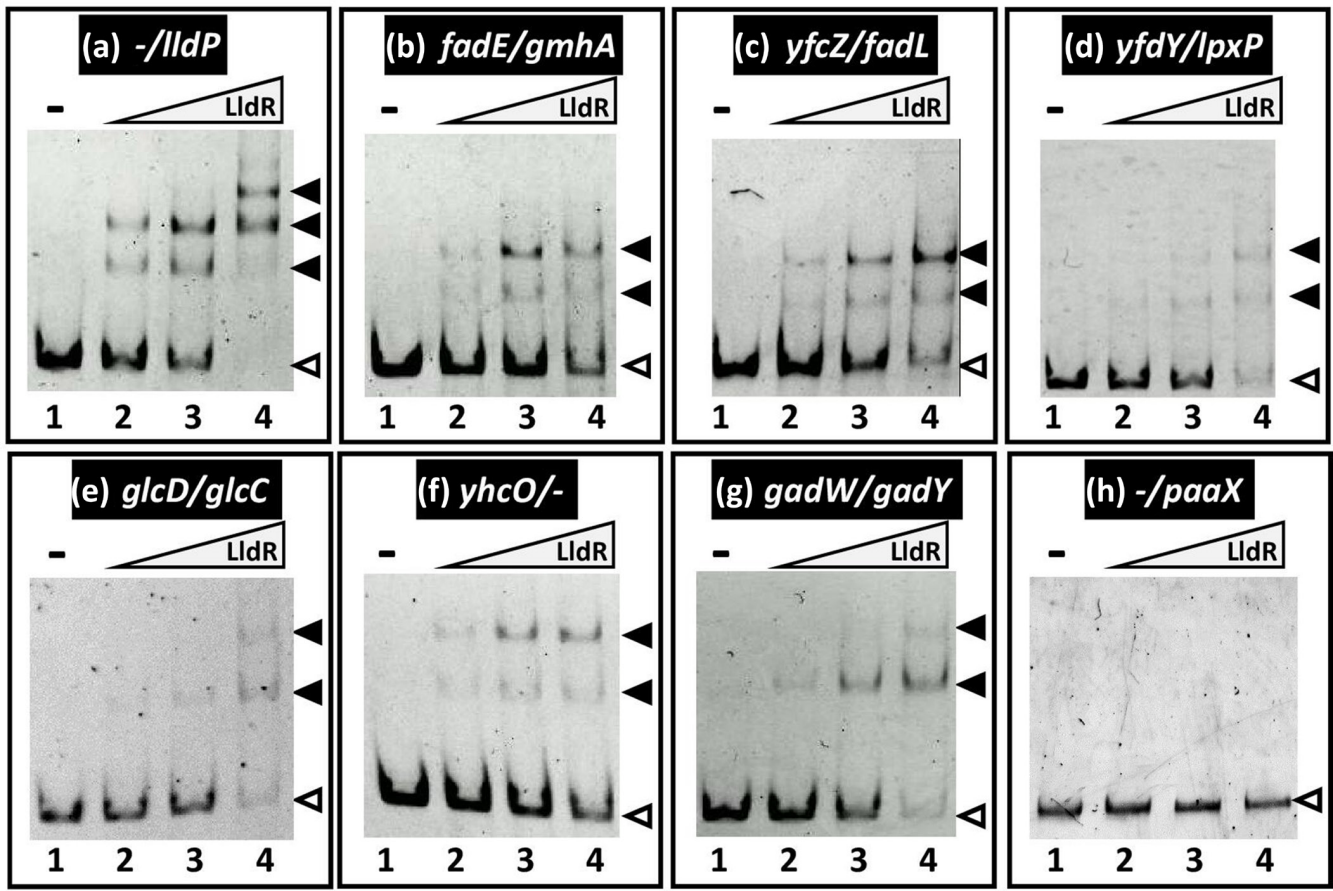
Gel shift assay of LldR-DNA complex formation. (**a-h**) Purified LldR was mixed with 0.5 pmol of each target DNA probe to the LldR-binding regions shown in Fig. 1. Concentration of LldR added (in pmol): lane 1, 0, lane 2, 20, lane 3, 80, and lane 4, 320. Filled triangles indicate the LldR-DNA probe complex, whereas open triangles indicate free probes. The promoter region of *paaX* was used as a negative control because no binding of LldR was observed in the gSELEX-chip screening.

To confirm the binding activity of LldR *in vitro* to the target sites predicted based on gSELEX screening we carried out a gel shift assay to detect LldR-target DNA complexes. *lldP* promoter region was prepared as a known target of LldR and used to confirm the accuracy of the assay system. This reference probe was mixed with increasing concentrations of purified LldR, and the probe-LldR mixtures were directly subjected to PAGE. It has been reported that the *lldP* promoter has two LldR-binding sequences [5]. The probe formed several LldR-DNA complexes in an LldR concentration-dependent manner (Fig. 2a). Next, six probes were prepared for the newly identified targets (Table 1) and subjected to gel shift assay under the same conditions. All six probes formed LldR-concentration-dependent LldR-probe DNA complexes (Fig. 2b–g). Among these LldR targets, the binding activity of LldR to the *lldP* promoter was the highest, and the promoter formed a complex with LldR at low concentrations of LldR, consistent with the results showing that the highest intensity was in *the lldP* promoter region in the gSELEX-chip (Fig. 1). By contrast, the *paaX* promoter region, a reference DNA added as a negative control, not detected in gSELEX screening, did not form LldR-DNA complexes under the same conditions (Fig. 2h). These results indicated the specific binding of LldR to all seven LldR target sequences (Table 1).

### Consensus recognition sequence of LldR

**Fig. 3.**
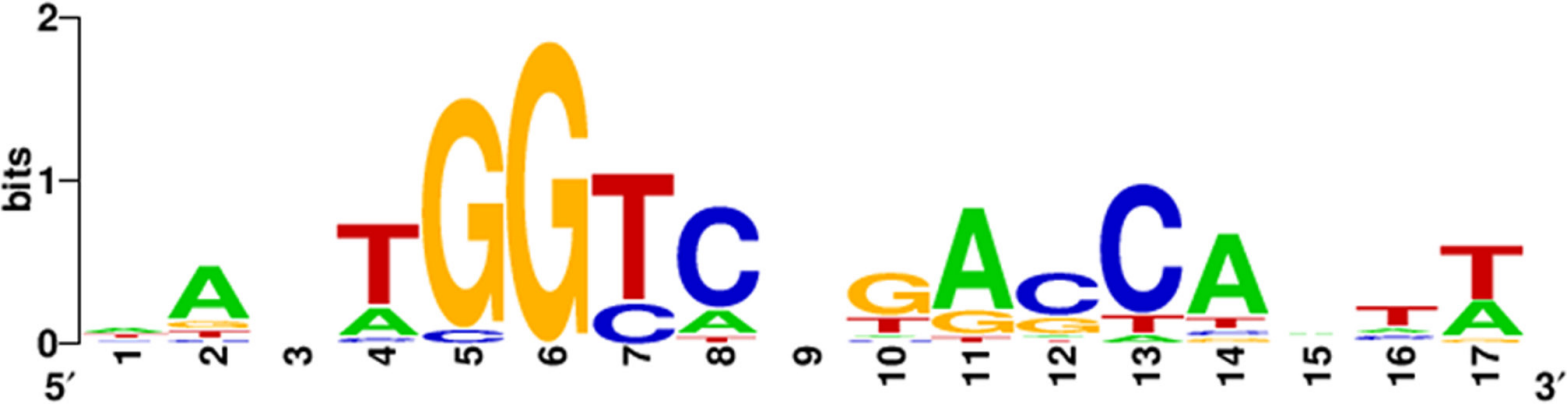
The palindromic consensus sequence of LldR-box. Sequences of all probes with LldR-binding activity were analysed using the MEME Suite (https://meme-suite.org/meme/) (see Table 1). WEBLOGO (http://weblogo.berkeley.edu/logo.cgi) was used to perform matrix construction.

Multiple LldR-probe complexes were observed for all tested probes in gel shift assay, suggesting the presence of multiple LldR-binding sequences in the LldR target regions. The resolution of the tiling array used in this study is approximately every 100 bp for the genome sequence; however, the spacing is biassed in some regions. In addition, the genomic DNA fragments used are 200–300 bp, and targets with multiple binding regions are detected as peaks spanning multiple probes. Therefore, to analyse the LldR binding sequence in the target region, a collection of 500 bp sequences from the LldR targets was analysed using the MEME Suite programme [42]. We identified a 17-bp-long palindromic sequence, AAnTGGTCnGACCAnTT (Fig. 3), which was present in two sets in all LldR-binding regions (Table 1). Furthermore, the identification of both LldR-box sequences in the *lldP* promoter region using MEME was consistent with the two LldR-binding sequences in the *lldP* promoter region reported by Aguilera *et al*. [5]. Thus, we concluded that all the LldR-binding regions identified by the gSELEX-chip could bind two molecules of LldR, and this 17 bp LldR box sequence is required to ensure tight binding of LldR.

### Regulatory role of LldR in the expression of the target genes *in vivo*

**Fig. 4.**
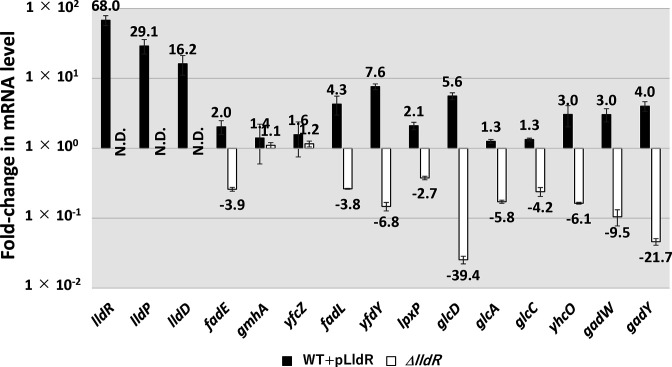
*In vivo* influence of *lldR*-deletion or LldR-overexpression on transcription level of LldR targets using RT-qPCR. Wild-type *E. coli* BW25113, its lldR-deleted mutant JW3579, the wild-type strain harbouring the LldR overexpression vector, and its empty vector were grown in the M9 medium with 0.2 % CAA at 37 °C. Total RNA was extracted from exponential phase *E. coli* cells (OD_600_=0.4), and subjected to RT-qPCR analysis. The y-axis represents the relative level of mRNA of each LldR target gene between the wild-type harbouring LldR overexpression vector and empty vector (black bar) as well the relative level between the wild-type and *lldR* mutant (white bar); the ratio of 16S rRNA is set as an internal control between the compared strains. Each experiment was repeated at least three times, and the average means are shown. ND indicates it was not detected as it was below the detection limit.

To examine the possible influence of LldR on the target promoters detected *in vitro* based on LldR-binding activities, we performed RT-qPCR analysis to determine the mRNA levels *in vivo* for each of the predicted LldR target genes in the presence, absence, or overexpression of LldR (Fig. 4). Total RNA was prepared from cells of wild-type *E. coli* K-12, its *lldR*-deleted mutant, the wild-type strain harbouring the LldR overexpression vector, or the empty control vector, grown in M9 minimal medium with 0.2 % CAA, and the mRNA levels of individual LldR target genes were measured. Total RNA was purified during the exponential phase and subjected to RT-qPCR.

The mRNA level of LldR itself was confirmed to be increased by approximately 70-fold in the LldR-overexpressing vector carrying the wild-type strain compared to the control strain, which harboured an empty vector. Furthermore, as a result of LldR overexpression, the genes that constitute the *lldPRD* operon, in which LldR has been reported to act as an activator, were found to be activated by LldR as expected, with mRNA levels of *lldP* and *lldD* increasing 16- and 29-fold, respectively. Under the same conditions, the mRNA levels of the newly identified LldR target genes identified by gSELEX were increased more than two-fold by the overexpression of LldR in *fadE, fadL, fadL, yfdY, lpxP, glcD, yhcO, gadW, and gadY* (Fig. 4). By contrast, the mRNA levels of these genes decreased by more than two-fold in the *lldR*-deficient strain compared to those in the wild-type strain. In addition, only a slight increase was observed in the mRNA levels of *glcA* and *glcC* upon overexpression of LldR, whereas a four-fold decrease was observed in the *lldR-*deficient strain. These results suggest that LldR acts as an activator not only of the lactate metabolism operon *lldPRD* but also of *fadE, fadL* and *lpxP,* which are involved in fatty acid and membrane metabolism; the *glcDEFGBA* operon and its regulator *glcC*, which are involved in glycolate metabolism; *gadW* and *gadY*, which are involved in acid tolerance; and *yfcZ* and *yfdY*, whose functions are unknown.

### Search for effectors controlling LldR activity *in vitro* and *in vivo*

**Fig. 5.**
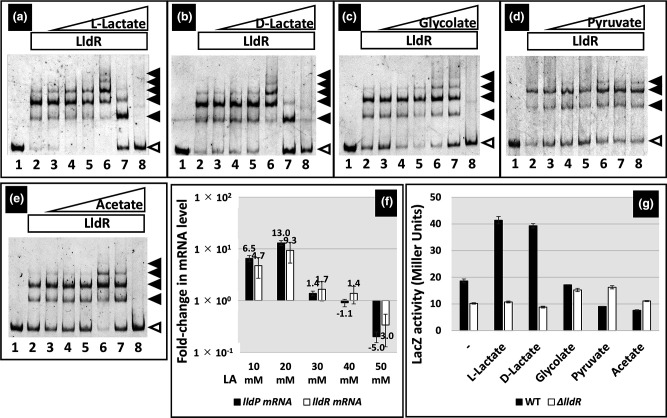
Search for inducers of LldR. (**a-e**) Gel shift assay of LldR–DNA complex formation and influence of each organic acid. Purified LldR was mixed with 0.5 pM each of a DNA probe corresponding to the promoter sequence of *lldP* gene. In the presence of 320 pmol LldR (lanes 2 to 8), each organic acid ((**a**), l-Lactate; (**b**), d-Lactate; (**c**), Glycolate; (**d**), Pyruvate; and (**e**), Acetate) was added (in mM): lanes 1 and 2, 0; lane 3, 0.1; lane 4, 0.5; lane 5, 1; lane 6, 5; lane 7, 10; and lane 8, 50. Filled triangles indicate the LldR-DNA probe complex, whereas open triangles indicate free probes. (**f**) The transcription level of mRNA of *lldPRD* operon in various concentrations of l-Lactate in the wild-type strain. The strain was grown in the M9 medium with 0.2 % CAA at 37 °C. When cell growth reached an OD_600_ of 0.2, the various concentrations of l-Lactate (LA) was added and continued the cell growth. Total RNA was prepared from the strain when reaching an OD_600_ of 0.4 and was subjected to RT-qPCR analysis. Each mRNA level was calculated as a ratio to that in the absence of l-Lactate. Each experiment was repeated at least three times, and the average means are shown. (**g**) Reporter assay of the *lldP* promoter. Reporter assay of the *lldP* promoter was carried out using the *lacZ* reporter encoding *β*-galactosidase. Single copy *lacZ* gene reporter strains containing *plldP-lacZ* was constructed in wild-type strain. The strain was grown in the M9 medium with 0.2 % CAA at 37 °C. When cell growth was reaching an OD_600_ of 0.2, 20 mM of each organic acid was added and continued the cell growth. *β*-Galactosidase was measured after reaching an OD_600_ of 0.4. Each experiment was repeated at least three times, and the average means are shown.

To date, it has not been observed that LldR changes its affinity with target DNA via effectors. Therefore, we reevaluated the effectors of LldR *in vitro* and *in vivo*. To identify a possible inducer effector (or effectors) for activating the *lldPRD* operon (the major regulatory target of LldR), we first tried to identify metabolites affecting the binding of LldR to the *lldP* promoter as a probe *in vitro*. We tested various concentrations of l-lactate, d-lactate, glycolate, and pyruvate as substrates or products of the LldR regulon (Fig. 5). LldR-probe complex formation did not change significantly at concentrations l-lactate and d-lactate below 1 mM, but was enhanced when the concentrations were raised to 5 mM (Fig. 5a, b). A further increase in their concentration resulted in the dissociation of LldR from the probe. A slight effect was observed for glycolic acid at the range of concentrations tested (Fig. 5c), and no effect was observed for pyruvic acid (Fig. 5d). Acetic acid was also tested as an alternative organic acid, and similar effects were observed with l-lactate and d-lactate (Fig. 5e).

Next, to observe the effect of these organic acids on the regulation of LldR *in vivo*, firstly, we measured the mRNA levels of *lldP* and *lldR* in the wild-type strain by RT-qPCR in the presence of different concentrations of l-lactate. As a result, the mRNA levels increased with the addition of 20 mM l-lactate, but at higher concentrations, the mRNA levels decreased, and at 50 mM, mRNA levels were lower than in the absence of l-lactate (Fig. 5f). As shown in the next section, this may be because the addition of high concentrations of l-lactic acid is stressful to the cells. The effect of organic acids was significant at a concentration of 20 mM. We carried out an *lldP* reporter assay in wild-type and *lldR*-deletion mutants. To identify the specificity of the effectors of LldR activation, we measured the activity of the *lldP* promoter in the presence of 20 mM of these organic acids (Fig. 5g). In the wild-type strain, the *lldP* promoter was two-fold more activated than in the *lldR*-mutant strain and was more activated in the presence of l-lactate or d-lactate. By contrast, in *the lldR* knockout mutant, *the lldP* promoter was not activated by l-lactate or d-lactate. On the other hand, no significant induction was observed with the addition of glycolate, pyruvate, or acetate. These *in vitro* and *in vivo* results confirmed that the effective effectors of LldR are l-lactate and d-lactate. Therefore, the effects of high concentrations (50 mM) of l-lactate, d-lactate, glycolate, and acetic acid may have non-specifically inactivated proteins or caused cell damage because of general acidic effects (Fig. 5a–c, e, f).

### Effect of lactate on *E. coli* growth and the effect of LldR

**Fig. 6.**
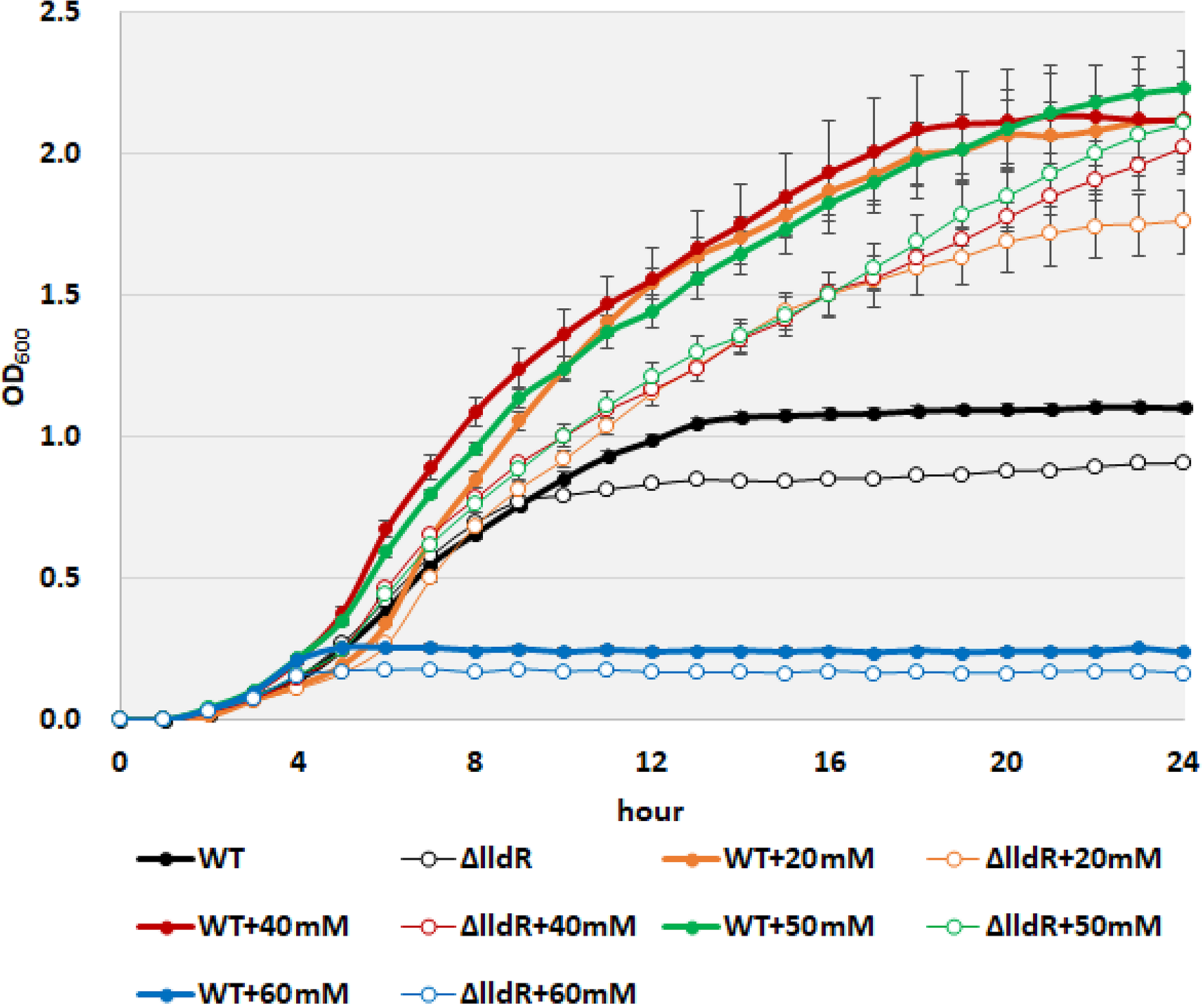
Influence of l-lactate on growth of *E. coli* K-12. *E. coli* wild-type BW25113 and its *lldR*-deleted mutant JW3579 were grown in M9 with 0.2 % CAA. When cell growth reached an OD_600_ of 0.2, the various concentrations of l-lactate was added and continued the cultivation. Cell growth was monitored during 24 h by measuring the OD_600_. The average of triplicate experiments is shown.

In *E. coli *, the response of the expression of the *lldPRD* operon to lactate has been reported [1, 5], but the effect of lactate on cell growth are not well understood. Therefore, the influence of l-lactate on cell growth was examined in wild-type *E. coli* and its *lldR* mutant grown in M9 minimal medium with 0.2 % CAA, the same medium used in the gene expression analysis. Under these conditions, the growth curves of the wild-type and *lldR* deficient strains did not differ during the exponential growth phase until approximately 9 h, but the final cell density was slightly higher in the wild-type strain (1.1 for the wild strain and 0.9 for the deficient strain) (Fig. 6). When 20 mM l-lactate was added to the medium, the final cell density increased to approximately 2.1 and 1.8 in the wild-type and deficient strains, respectively, indicating that lactate was used as a carbon source. When the lactate concentration was further increased to 40 mM, the onset of growth initiation came earlier in the wild-type strain, but little effect was seen on the *lldR* deficient strain. Increasing l-lactate concentration to 50 mM led to growth reduction in the wild-type strain, and the same positive effect was observed at 40 mM. However, when the l-lactate concentration was increased to 60 mM, significant inhibition of cell growth was observed, which was more pronounced in the *lldR* deficient strain than in the wild-type strain. These results suggest that l-lactate acts as a growth enhancer for *E. coli* at concentrations up to 40 mM in the medium but has additional stress effects at concentrations above 50 mM (Fig. 6).

### Regulatory role of LldR on lactate as stress

Since the effect of lactate on *E. coli* as a simple acid stress is already clear, we attempted to clarify the regulatory role of LldR against this response. Among the newly identified LldR-target genes using gSELEX screening, *gadW* encodes a regulator of the glutamate-dependent (GAD) system [47, 48], the principal acid resistance system is known to be AR2 [21], and *gadY* encodes non-coding RNA, a positive regulator of *gadW* and *gadX* by stabilising their transcript products [49, 50]. The primary roles of GadW and GadX are the activation of *gadA* and *gadB*, which encode glutamate decarboxylases and *gadC*, the l-glutamate: 4-aminobutyrate antiporter, which confers tolerance to acidic conditions [51, 52]. Northern blot analysis was performed to observe the mRNA levels and the effects of LldR and l-lactate on the transcript levels of these direct LldR target genes, the regulators and their target genes involved in the GAD system.

**Fig. 7.**
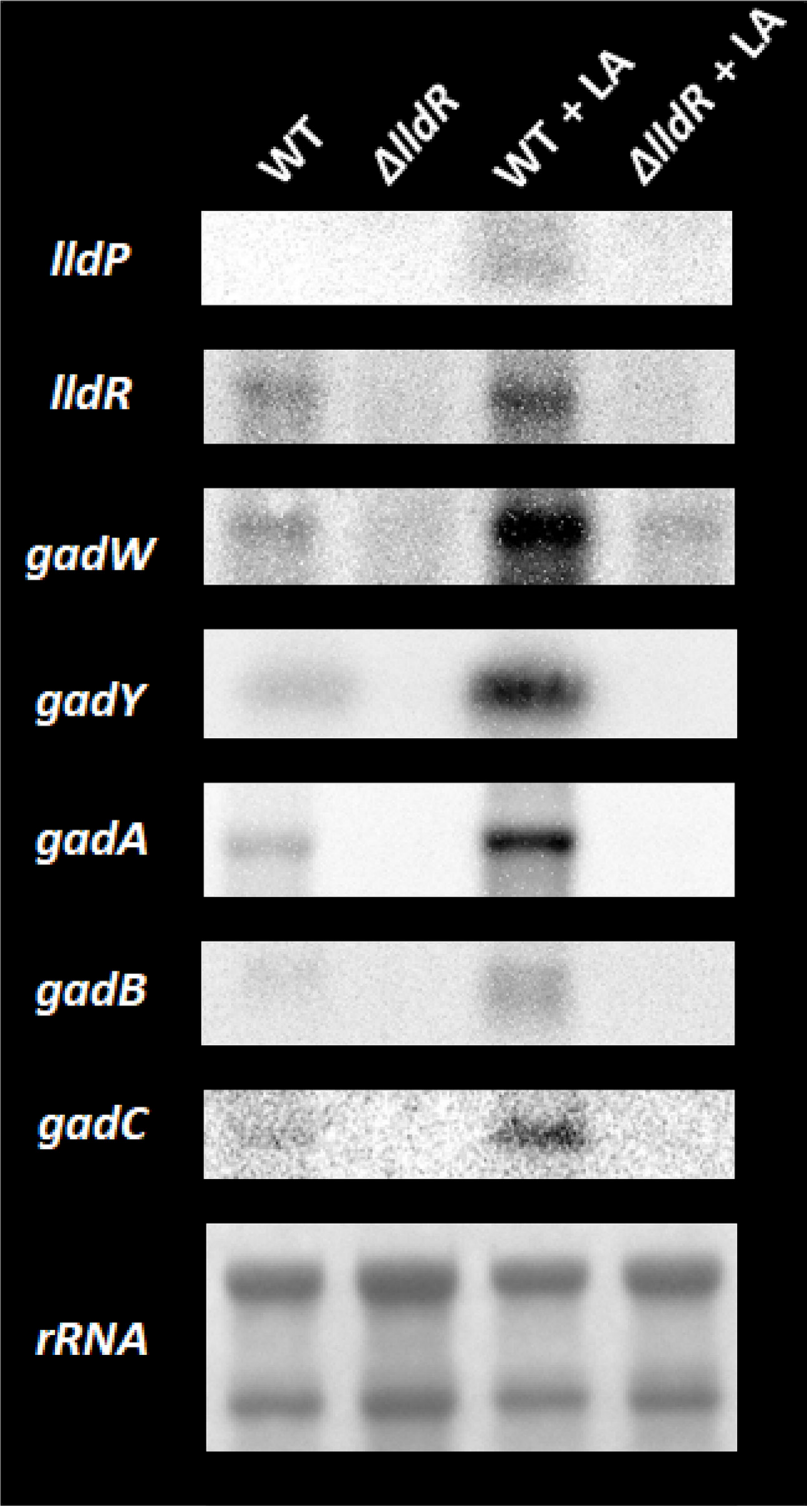
*In vivo* influence of l-lactate on transcription level of the LldR targets involved in GAD system by Northern blot analysis. *E. coli* wild-type BW25113 and its *lldR*-deleted mutant JW3579 were grown in the M9 medium with 0.2 % CAA at 37 °C. When cell growth was reaching an OD_600_ of 0.2, 20 mM of l-Lactate (LA) was added and continued the cell growth. Total RNA was prepared from the strain when reaching an OD_600_ of 0.4 and was subjected to Northern blot analysis. DIG-labelled hybridization probes are shown on the left side of each panel. The amounts of total RNA analysed were examined by measuring the intensity of ribosomal RNAs.

Two sets of wild-type *E. coli* and its *lldR* mutant were grown in M9 minimal medium with 0.2 % CAA, and when the OD_600_ reached 0.2, 20 mM of l-lactate was added to one set. Total RNA was prepared from the strains when reaching an OD_600_ of 0.4 and subjected to Northern blot analysis (Fig. 7). In the absence of lactate, target transcripts were barely detectable in the *lldR* deficient strain, whereas low-intensity bands were detected in the wild-type strain. In the *lldR* deficient strain, the transcripts were barely detectable in the presence or absence of l-lactate. By contrast, clear bands were detected in the wild-type strain in the presence but not in the absence of l-lactate. l-Lactate at a concentration of 20 mM is considered virtually acid stress-free for *E. coli *, which is consistent with the fact that no induction of the GAD system was observed in the *lldR* deficient strain in the Northern blot analysis even when l-lactate at this concentration was added. Taken together, these results indicate that l-lactate transcriptionally activates the GAD system in an LldR-dependent manner.

### The physiological role of LldR on lactate as stress

**Fig. 8.**
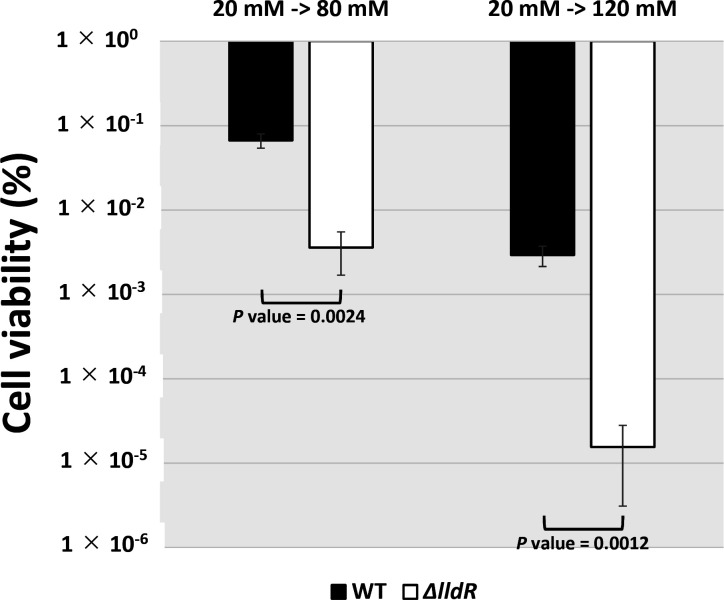
Sensitivity test against l-lactate. Survival test for wild-type *E. coli* BW25113 and its *lldR*-deleted mutant JW3579. The relative level of sensitivity represents the mean±SD of four independent experiments. Statistical significance was analysed using the Student’s t-test with multiple comparisons’ correction and are represented by line under bars with associated *P* values.

The newly identified GAD system, the major acid tolerance mechanism in *E. coli *, is induced by l-lactate in an LldR-dependent manner. Next, we examined the physiological role of LldR. To test the role of LldR in acid stress resistance against lactate, *E. coli* wild-type and its *lldR* deleted strains were inoculated in M9 medium with 0.2 % CAA, and 20 mM l-lactate was added from the start of the culture for pre-induction of the LldR regulon. Next, when the OD_600_ reached 0.3, l-lactate was added to the medium’s final concentrations of 80 mM or 120 mM, and incubation was continued for 30 min. The inoculated medium was then spread on an agar plate, and the number of colonies formed was counted and measured as the number of viable cells. The results showed that the survival rates of the wild-type strain were 7 and 0.4 % in 80 or 120 mM l-lactate, respectively, whereas those of the *lldR* deficient strain were 0.3 and 0.002 %, respectively (Fig. 8). The cell viability ratio of wild-type to deficient strains was more than 20-fold under 80 mM l-lactate conditions and more than 200-fold under 120 mM l-lactate conditions, indicating that *lldR* deficiency increased sensitivity to lactate as acid stress.

**Fig. 9.**
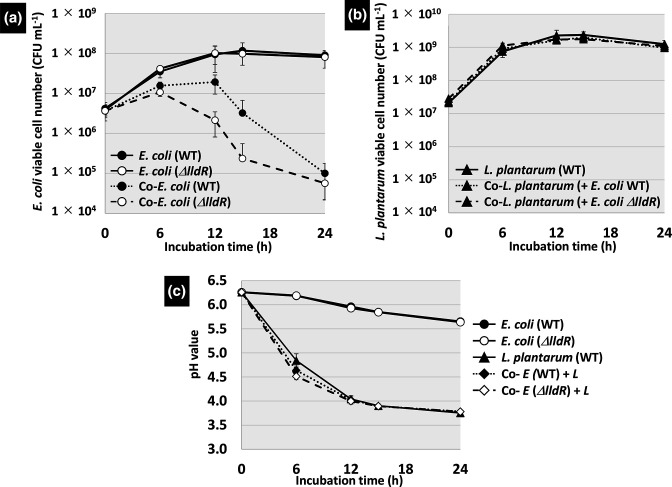
Growth of wild-type *E. coli* and its *lldR*-deficient strains in mono- and co-culture with *L. plantarum* in MRS medium. (**a**) Growth of *E. coli* mono- and co-cultures. (**b**) Growth of *L. plantarum* mono- and co-cultures. (**c**) Change in pH of MRS medium during mono- and co-culture. Sampling was done at 0, 6, 12, 15, and 24 h. The relative level of sensitivity represents the mean±SD of three independent experiments.

In the human intestinal environment, where *E. coli* naturally lives, the effects of probiotic foods ingested by humans are particularly pronounced [19, 20]. *E. coli* is exposed to lactic acid produced by lactic acid bacteria [16, 17]. Therefore, we examined the role of LldR in the coexistence of *E. coli* and *Lactiplantibacillus plantarum *. When the wild-type and *lldR*-deficient *E. coli* were mono-cultured in MRS medium under anaerobic conditions, there was no difference in growth, and both reached 10^8^ c.f.u. ml^−1^ in the stationary phase (Fig. 9a). The pH of the medium during this period gradually decreased from pH 6.3 to 5.6 as the cells grew (Fig. 9c). When *E. coli* was co-cultured with *L. plantarum *, the growth of both strains of *E. coli* was suppressed, but a severe effect was observed in *the lldR*-deficient strain (Fig. 9a). In the wild-type strain, the viable bacterial count decreased to approximately 3×10^6^ at 15 h. By contrast, in the *lldR*-deficient strain, viable counts dropped to approximately 2×10^6^ at 12 h and 2×10^5^ at 15 h. At 24 h, the viable counts for both strains dropped to 10^5^, but the negative effect of co-culturing with lactic acid bacteria on survival was greater for the *lldR*-deficient strain. The pH of the medium during this co-cultivation was significantly lower, being around pH 4.6 at 6 h, but after 12 h, when the viable *E. coli* count had decreased, the pH was 4.0, and after 24 h, the pH dropped to 3.8 (Fig. 9c). The decrease in pH of the medium was almost the same as that in the *L. plantarum* monoculture. Under this co-cultivation condition, *E. coli* did not affect the viable counts of *L. plantarum* (Fig. 9b). These results suggest that LldR contributes to tolerance to the decrease in environmental pH caused by the lactic acid produced by lactic acid bacteria.

## Discussion

### LldR is a local TF for adaptation to lactate

*E. coli* K-12 contains approximately 300 TFs [46, 53]. We have been involved in a systematic search of regulatory targets for all TFs using gSELEX screening [26]. To date, we have succeeded in listing regulatory target promoters, genes, and operons for approximately half of *E. coli* TFs [53; and TEC database: https://shigen.nig.ac.jp/ecoli/tec/top/]. One interesting finding is that the number of regulatory targets is highly variable, ranging from single targets, referred to as single-target TFs [43, 54], to more than 5000, referred to as global TFs [27]. Prior to this study, only one regulatory target of LldR was identified as *the lldPRD* operon [1], thus, LldR was thought to be a single-target regulator. After gSELEX screening, the number of LldR targets was found to be 12 gene(s) or operons in *the E. coli* genome (Fig. 1 and Table 1), indicating that LldR is a local TF which has a regulatory role for a small number of specific targets [27].

In this study, we observed the influences of some organic acids on the activity of LldR *in vitro* and *in vivo*, and concluded that l-lactate and d-lactate are influential effectors of LldR in *E. coli* (Fig. 5). Interestingly, in the presence of a low concentration of effectors, LldR binding to the target promoters was stimulated, but under an excess concentration, LldR was dissociated (Fig. 5a, b, f). This may imply a function similar to that of a safety valve against l-/d-lactate acid stress caused by excess lactate uptake into the cell through the activation of the lactate permease encoding *lldP,* which is one of the major roles of LldR. The affinity of LldR differed by target, with higher affinity for the *lldPRD* operon using lactate as a carbon source compared to the other promoters involved in acid tolerance (Figs 1 and 2). This difference in affinity of LldR to each promoter suggests that LldR can respond to lactate concentration by using lactate as a carbon source at low concentrations, subsequently inducing acid tolerance as the concentration increases, and then stopping uptake at higher concentrations. This is also supported by the effect of lactic acid on cell growth, in which low concentrations of lactic acid had a positive effect on cell growth, whereas high concentrations had a negative effect (Fig. 6). The unknown functional genes, *yfdY* and *yhcO*, were also found to be activated by LldR (Fig. 4). These genes may also be involved in lactate adaptation in *E. coli *. Future functional elucidation of these genes will be helpful for further understanding the lactate response.

### Novel regulatory role of LldR on lactic acid as a carbon source

Although the *lldPRD* operon, which converts lactate to pyruvate for uptake, has hitherto been the sole target of LldR, this study revealed that the *glcDEFGBA* operon and its regulator, *glcC*, are also targets of LldR as activators (Figs 2 and 4). The *glc* operon is involved in glycolate and glyoxylate degradation and consists of *glcDEF* for glycolate dehydrogenase, *glcG* for putative haem-binding protein, *glcB* for malate synthase, and *glcA* for glycolate: proton symporter. The glycolate dehydrogenase complex, GlcDEF, oxidises glycolate to glyoxylate, and reacts at a similar reaction rate to d-lactate and a slower reaction rate with l-lactate [55]. In addition, GlcA, a transporter of glycolate, has been reported to take up lactate, although it is less active than glycolate [3]. Thus, the *glc* operon can metabolise glycolate and lactate, suggesting that lactate metabolism is more efficient because it is activated by the lactate response-sensing TF LldR in addition to the *lld* operon. Recently, we found that these *lld* and *glc* operons are also controlled by the transcriptional regulation by pyruvate-sensing TF PdhR [8]. Taken together, we propose that the metabolism of organic acids is regulated by a comprehensive transcriptional regulatory network that reflects the intracellular concentrations of their substrates and products, which are cross-regulated by TFs that control the original metabolic pathway.

### Novel regulatory role of LldR on acid stress induced by lactic acid

This study identified a set of genes involved in acid stress tolerance as new members of the LldR regulon (Fig. 1 and Table 1). The first group of genes is *gadW* and *gadY*, regulators of the GAD system, known as AR2 [21]. These regulators were activated by LldR (Figs 4 and 7), suggesting that GadW and GadX regulate the AR2 gene cluster, and by regulating these regulators, AR2 is used to achieve acid tolerance. It was confirmed that representative genes of AR2, such as *gadA, gadB,* and *gadC*, were activated in the presence of lactate and that regulation was LldR-dependent (Fig. 7). The second group of genes is involved in membrane properties and includes *fadE* (acyl-CoA dehydrogenase)*, fadL* (long-chain fatty acid outer membrane channel), and *lpxP* (palmitoleoyl acyltransferase). In bacteria, acid stress has been reported to stress membranes [24], e.g. to affect the activity of ATPase, an enzyme responsible for cellular energy mediation [56, 57]. Lipid metabolism has also been shown to enhance acid tolerance. For example, LpxP, the target of LldR, increases the amount of palmitoleate in lipid A, an unsaturated fatty acid, as a constituent fatty acid of the membrane instead of lauric acid, a saturated fatty acid [58], and has been reported to be enhanced when the amount of structurally flexible unsaturated fatty acids increases [56, 57, 59]. It has also been reported that *lpxP* is induced by acid stress, not only by lactate but also by hydrochloric or acetic acid [15]. This study also revealed that in addition to its effect on gene expression in response to lactate, LldR plays an important role in conferring acid resistance to *E. coli *. The effect of *lldR* on the survival rates of *E. coli* in the presence of high lactate concentrations (80 mM and 120 mM) was observed (Fig. 8); however, since these concentrations were not physiologically appropriate for a healthy colonic environment, it was next validated in co-culture with lactic acid bacteria (Fig. 9), showing the important role for *lldR* in both scenarios. Several studies on maintaining a healthy human intestinal environment have reported the death or growth inhibition of *E. coli* by co-cultivation with lactic acid bacteria [19, 20]. *L. plantarum* has been reported to produce both l- and d-lactate [60]. As shown in Fig. 5, LldR responds to both l- and d-lactate and induces the LldR regulon, which allows *E. coli* to adapt to lactic acid stress.

**Fig. 10.**
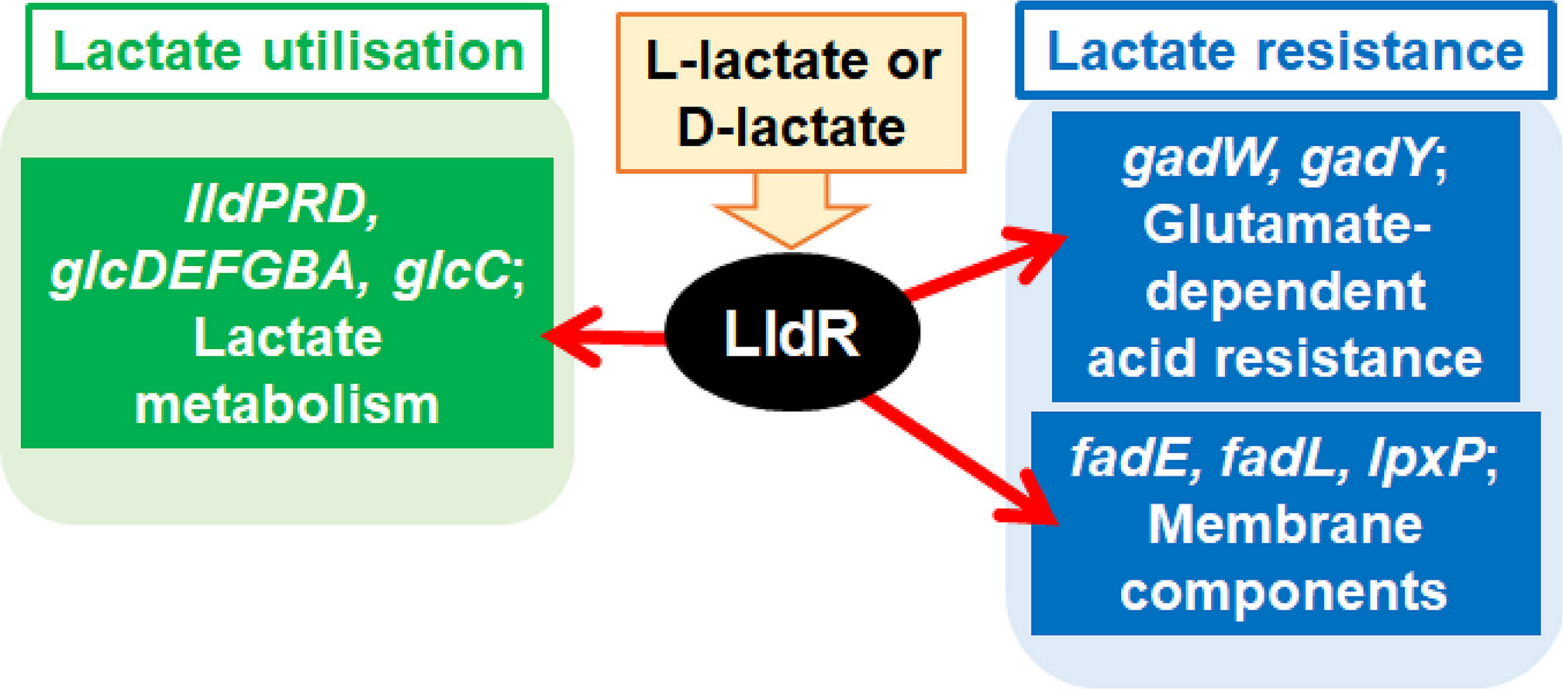
Model of regulatory network of lactate-sensing transcription factor LldR in *Escherichia coli* K-12 genome.

We propose that LldR is an l-/d-lactate-sensing TF for lactate utilisation as a carbon source and resistance to lactate as a form of acid stress in *E. coli* (Fig. 10). The findings of this study will be useful for understanding how enteric bacteria utilise and tolerate lactate in the intestine.

## Supplementary Data

Supplementary material 1
