# Supplementary material for: Expanded roles of lactate-sensing LldR in transcription regulation of the Escherichia coli K-12 genome: lactate utilisation and acid resistance
Source: Microb Genom. 2023 May 23;9(5):mgen001015. doi: 10.1099/mgen.0.001015 (PMC10272880; doi:10.1099/mgen.0.001015)
Supplement: Supplementary material 1 [file mgen-9-1015-s001.pdf]

Table S1. Primers used in this study.

(a) For gel shift assay

name	sequence
fadE/gmhA-F	TAAAGCTGTCTGCTAACAGG
fadE/gmhA-R	AACGAAAAGCCCCTTACTTG
yfcZ/fadL-F	TTGATTTCCTCTGTATGTGC
yfcZ/fadL-R	GACCATAACCTCAATGATTTAT
yfdY/lpxP-F	AGAAACATCCATAAATTAATCATAG
yfdY/lpxP-R	AGTTACCTTCTGTAGATACGAATT
glcD/glcC-F	AAGACGCTCTTCGTACAAG
glcD/glcC-R	TCCCGGACCTCGTGCAC
yhcO/--F	TTTTGCTGATGAATAAGTTCCC
yhcO/--R	CTGTTGCCACCGTCACGT
gadW/gadY-F	AGAATACTCTCGTTATGCAGC
gadW/gadY-R	AAGTTAAATATAACTTTTACTGGAAATAAG
-/lldP-F	CAGGAGATGACTGGGTTTTC
-/lldP-R	TGCAGGTCTCCTGGAGTCCA
-/paaX-F	GATTTTCAACCAGATATGATCA
-/paaX-R	TAACAATTTCAGGATAGAAATGAT

(b) For RT-qPCR analysis

name	sequence
fadE-F	ACAATGACGTCAACGCGTTC
fadE-R	TGCGAACTTTGTTGCTACCG
gmhA-F	CTGCAAACGTGATCAAAGCG
gmhA-R	ATTTTGCCGCCGTCTTTACC
yfcZ-F	TGACTGAAAAAGCCCGTAGC
yfcZ-R	TTCAGCTTCGCAGGCAAAAG
fadL-F	TTTGACCGCCCGACATTTTC
fadL-R	AAAGTGCATGTTCGGAACCC
yfdY-F	TTCTCGCCCTGTGTATTGTCTG
yfdY-R	AAATGAGTGCAGCGAGGATC
lpxP-F	ACTTTCGTTCACTCGGCATG
lpxP-R	CCGCGATTTTGCATTTGTGC
glcD-F	AAATGTGCGCCCAGTTCAAC
glcD-R	TGCATGGCACCAAATTCAGC
glcA-F	GGCCGAAAAATACCGAAACG
glcA-R	ACCACGCTCGAATGATTTGC
glcC-F	ACGTCGACTGTGTGAAAAGC
glcC-R	TGCGCCGTTTCAATAATCCC
yhcO-F	GTTCATCTGGGAGAGAAAACGC
yhcO-R	AAACGCAAATGCCCTTCCAG
gadW-F	TCGATGAGCTGACAGTTTGC
gadW-R	TCAAAAATCGCCGTCACCAG
gadY-F	ACAAAGTTTCCCGTGCCAAC
gadY-R	AAGAGGATAGTCTGCCGTCTC
lldP-F	TGAAAACCGGGCAGTTTGAC
lldP-R	AACCGACGATCAGCATTTGC
lldR-F	ATCGTCCAGCCGCTAAAAAC
lldR-R	TTTCTTTGTCGCCAGGTGTG
lldD-F	TTCCGTTTGCCCGATTGAAG
lldD-R	AAAACCAGCGTCGAACAACC
rrsA-F	TGCATCTGATACTGGCAAGC
rrsA-R	TACGCATTTCACCGCTACAC

(c) For promoter assay

name	sequence
plldR-F	CCGGAATTCCAGGAGATGACTGGGTTTTC
plldR-R	CGCGGATCCTGCAGGTCTCCTGGAGTCCA

(d) For Northern blot analysis

name	sequence
lldP-F	ATTCTGTTTGCTGCACTGCTCTC
lldP-R	GGAATCATCCACGTTAAGACATAAG
lldR-F	ATTGATGAAAAAAACCTGGAAG
lldR-R	GTGGTGTGAACAAAACTAAGGT
gadW-F	TTACTTACAAAATATTCGTCAGC
gadW-R	CACCATAATATTGTCGAAATG
gadY-F	ACTGAGAGCACAAAGTTTC
gadY-R	AAAAAAACCCGGCATAGG
gadA-F	GATTTCCGCTCAGAACTACTC
gadA-R	GGTTGTGGGAACTCATAGTTAC
gadB-F	AAGAAGCAAGTAACGGATTTAAG
gadB-R	TAATGGATAAATCCATCAATTTGT
gadC-F	CTTTTCATTAGTCTTCTTCCTGC
gadC-R	AAACCACCAACAGAGCTTAAG

DNA band patterns of genomic DNA fragments on PAGE

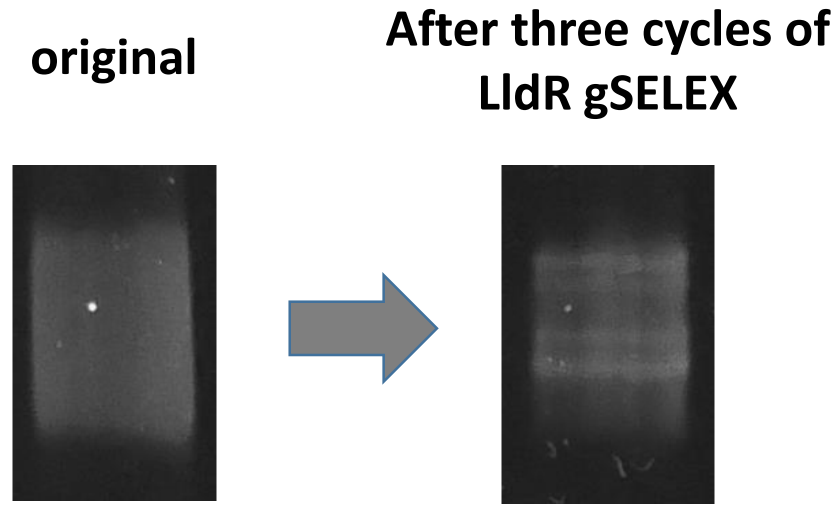

Figure S1. DNA band patterns of genomic DNA fragments. The SELEX cycle was repeated three times to enrich the LldR-binding sequences. The original DNA in gSELEX and the DNA after three cycles were each run on PAGE, and the DNA was stained with GelRed for observation.
